# Universal Suicide Screening in the Hospital Setting: Risk and Implications

**DOI:** 10.1007/s11414-026-09997-z

**Published:** 2026-04-27

**Authors:** Mairin Cotter, Dinan Noor, Kimberly S. Barrier, Kailey N. Clinton, John B. Britt, Cynthia W. Karlson

**Affiliations:** 1 Department of Psychology, University of Southern Mississippi, Hattiesburg, MS USA; 2 Department of Psychiatry and Human Behavior, University of Mississippi Medical Center, Jackson, MS USA; 3 Department of Pediatrics, University of Mississippi Medical Center, Jackson, MS USA; 4 Nursing Administration, University of Mississippi Medical Center, Jackson, MS USA; 5 Department of Pediatrics, Division of Hematology/Oncology, University of Mississippi Medical Center, 2500 North State Street, Jackson, MS 39216 USA

## Abstract

Suicide risk screening is a pivotal step in improving hospital safety and quality of care, with the potential to prevent suicide and facilitate mental health intervention. The Ask Suicide-Screening Questions (ASQ) is a brief, validated tool for pediatric and adult populations. This retrospective cross-sectional study sought to identify prevalence rates of suicide risk among pediatric and adult patients at an academic medical center in the Southeastern United States, examine demographic factors associated with ASQ outcomes, and compare suicide risk rates across presenting medical diagnoses. The ASQ was administered to patients 12 years and older upon admission to the ED or inpatient unit. Partial proportional odds modeling was used to examine associations between demographic factors and suicide risk. Chi-square analyses were used to identify differences in suicide risk across medical diagnoses. On the ASQ, 5% of pediatric patients (12–24 years) and 1% of adults (25–113 years) screened as potential risk, and 1% of both samples screened as imminent risk. Higher-risk groups included early adolescents (12–14 years), young adults (25–34 years), pediatric females, adult males, and adult patients with government insurance or no insurance The psychiatric/substance use category had significantly higher rates of suicide risk compared to other diagnostic categories. Among non-psychiatric pediatric patients, neurological, chronic condition, and general medical diagnoses exhibited higher potential risk than women's health, trauma, and orthopedic diagnoses. Results highlight need for universal suicide risk screening in hospital settings and indicate elevated risk among younger adolescents, young adults, and adults with government insurance or no insurance.

## Introduction

Suicide is a critical global public health concern, with an estimated 720,000 people dying by suicide worldwide each year.^1^ In the United States, suicide was the 11th leading cause of death in 2022, with an age-adjusted rate of 14.2 deaths per 100,000 and the highest attempts of suicide among individuals aged 18–25 years.^2,3^ Suicidal ideation, defined as thoughts of self-harm or ending one’s life, is a recognized predictor of suicide.^4^ Data from the National Center for Health Statistics Rapid Surveys System (RSS) indicated that 5.3% of U.S. adults reported suicidal ideation.^5^, Identifying and addressing suicidal ideation is crucial for preventing suicidal behaviors and improving quality of life.^6^ Suicidal ideation exists on a continuous spectrum, and can range from passive thoughts of wanting to die without intent or a plan to active suicidal ideation (e.g., a formulated plan and intent to die).^7,8^

The observed variability in suicidal ideation among individuals experiencing suicidal ideation underscores the importance of consistent suicide screening during hospital visits.^9,10^ In fact, data published from the Centers for Disease Control and Prevention (CDC) in August 2025 revealed that out of every 100,000 visits to the emergency department (ED), 151 visits were related to a suspected suicide attempt.^11^ The Joint Commission’s National Performance Goal (NPG) 08.01.01 mandates that all accredited hospitals assess patients for suicide risk, primarily focusing on psychiatric hospitals and patients with emotional or behavioral disorders in general hospitals.^12^ However, a significant number of non-psychiatric patients do not undergo suicide screening. For example, a 2025 study reported that out of 284 hospitals, 20.8% had not implemented universal suicide risk screening.^12^ Further, in a sample of patients who died by suicide, over 90% of patients had visited a medical provider within the prior year, and over a fifth of patients visited the ED within two months before their death.^13^ These patients often present with somatic complaints and do not disclose psychiatric symptoms unless specifically asked.^14^ Therefore, timely and effective suicide screening tools are essential for identifying at-risk individuals in healthcare settings.

The National Institute of Mental Health developed the Ask Suicide-Screening Questions (ASQ), a brief, validated tool designed to identify pediatric and adult patients at risk for suicide in medical settings, including the ED, medical hospitals, psychiatric hospitals, and outpatient care.^15^ The ASQ is one of the first suicide risk-screening tools developed specifically for young patients in the ED and is effective in identifying at-risk individuals across age groups.^16,17^ Prior studies have examined the predictive validity of the ASQ in either pediatric or adult patients separately.^16–19^ However, no studies to date have investigated the feasibility or utility of the ASQ for suicide risk screening across both pediatric and adult populations. This present study addresses this gap by examining suicide risk in both pediatric and adult samples through universal screening with the ASQ across a large hospital system. Specific aims were to (1) identify the prevalence rate of suicide risk (i.e., suicidal ideation and/or suicide attempt as measured through the ASQ) among both adult and pediatric patients seen at an academic medical center in the Southeastern United States, (2) examine demographic associations with ASQ screening outcomes, and (3) conduct a novel analysis to understand suicide risk across different presenting primary diagnosis categories.

## Methods

### Sample and setting

This retrospective study was self-certified as a quality improvement project to ensure high adherence to Joint Commission guidelines on suicide screening. This study took place at the only academic medical center in a Southeastern state that treats a racially diverse patient population from both rural and urban regions. Participants included pediatric (12 to 24 years) and adult patients (25 years and older) seen in the ED or admitted to the hospital. Data was examined from the initial year of universal screening (May 2023 to April 2024) in which all patients 12 years and older were administered the ASQ. During this time period, the medical center conducted a total of 76,924 ASQ screenings (27% pediatric; 73% adult).

### Measures

#### Ask Suicide-Screening Questions (ASQ)

The ASQ is a 4-item suicide risk screening measure that assesses for recent suicidal ideation and lifetime suicide attempts.^15^ The ASQ was initially developed for pediatric ED patients. Results from this development study showed high sensitivity (96.9%) and specificity (87.6%), as well as high negative predictive value (i.e., 99.7% of patients admitted for medical care and 96.9% of patients admitted for psychiatric care were correctly identified as not at risk).^20^ Further, a 2020 validation study demonstrated high sensitivity (100%), specificity (89%), and negative predictive value (100%) for adult medical/surgical patients.^16^ The ASQ consists of the following four yes/no questions:

1. In the past few weeks, have you wished you were dead?
2. In the past few weeks, have you felt that you or your family would be better off if you were dead?
3. In the past week, have you been having thoughts about killing yourself?
4. Have you ever tried to kill yourself?

If any of the four questions on the ASQ are answered “yes,” a fifth item is administered (“Are you having thoughts of killing yourself right now?”) to determine the risk acuity. For this present study, if a patient answered “yes” to any of the first four items and answered “yes” to the fifth item, they were identified as imminent risk (Acute Positive Screen). Patients who responded “yes” to any of the first four items and responded “no” to the fifth item were identified as potential risk (Non-Acute Positive Screen). The identifier of no risk was provided to patients who answered “no” to the first four screening questions.

### Procedures

Upon admission to the ED or hospital, a nurse administered the ASQ to determine suicide risk. If a patient arrived unconscious and could not be screened at admission, they were screened when they became conscious and lucid. For patients with cognitive or developmental delays, the screen was administered to their guardian or caregiver. For patients in psychiatric units who scored in the Acute or Non-Acute Positive range on the ASQ, the provider was notified and then administered necessary risk assessments (i.e., Brief Suicide Safety Assessment [BSSA]) to determine suicidal intent, plan, suicidal or self-harm behaviors, risk factors, and protective factors.^21^ For patients in medical units, the Suicide Risk Screen Protocol was initiated by the nurse. This protocol involved notifying the attending physician, requesting a psychiatry consult, 1:1 constant observation (if Acute Positive Screen), additional risk assessments (e.g., BSSA) by a psychiatry or psychology provider, and necessary suicide precautions (e.g., environmental risk checks). If a patient (regardless of initial risk) exhibited a change in behavior (e.g., self-destructive behavior, verbalizing thoughts of self-harm), the ASQ was re-administered. Responses on the ASQ were entered into the hospital’s Electronic Medical Record system (EPIC) by the nurse.

#### Demographics

Demographic (i.e., age, race, sex, time of year/season, insurance type), medical (i.e., admission unit, chief complaint, presenting primary diagnosis), and ASQ variables were extracted from EPIC. Using CDC guidelines, the pediatric sample was categorized into early adolescence (12–14) and late adolescence/early adulthood (15–24) and the adult sample was categorized into young adulthood (25–34), middle adulthood (35–64), older adulthood (65–84), and late adulthood (85 +).^2^ Race was categorized into White, Black, Hispanic/Latine, Indian American, Pacific Islander/Hawaiian, and Multiracial groups.^22^ However, given the small amount of patients in minority categories, for statistical analyses, race was consolidated into White, Black, and other/minority groups. Presenting primary diagnosis was coded into seven categories based upon the primary diagnosis provided in each patient’s chart: orthopedic (e.g., fracture), psychiatric/substance use (e.g., psychosis, overdose, suicidal ideation), women’s health (e.g., labor and delivery, breast mass, vaginal bleeding), chronic condition (e.g., patients being seen for treatment of chronic illnesses such as sickle cell disease, cancer, or diabetes), neurological (e.g., seizure, stroke), general medical (e.g., chest pain, appendicitis, kidney stones), and motor vehicle collision (MVC)/gunshot wound (GSW)/penetrating trauma (e.g., stab wound, traumatic brain injury). The patient’s listed chief complaint was also utilized to inform primary diagnosis categorization.

### Statistical analysis

All analyses were performed separately for the pediatric and adult samples using IBM SPSS Statistics 30.0 and R Studio Version 2025.09. The percentage of missing data for demographic variables, primary diagnosis, and ASQ outcome was examined for both samples. Pairwise deletion was used for variables that had less than 10% of missing data (age, sex, race, insurance, season, ASQ outcome). Thirty-six pediatric patients (< 1%) and 139 adult patients (< 1%%) were not administered the ASQ due to lack of consciousness, disorientation, or health care provider was unable to administer due to an undocumented reason. 16% of pediatric patients and 9% of adult patients did not have a primary diagnosis listed in EPIC. These data were classified as Not Missing at Random (NMAR; e.g., due to administrative error) and was handled using pairwise deletion. Descriptive statistics characterized demographic and medical variables of the pediatric and adult populations. Chi-square analysis was utilized to compare demographic variables (i.e., age, race, sex, insurance type, season) between the three suicide risk levels. To examine the association between demographic variables and ASQ outcome, an ordinal logistic regression was first considered for the pediatric and adult sample. However, results from the Brant test suggested a violation of the proportional odds assumption for the omnibus model (*p* = 0.05) and insurance status (*p* = 0.04) in the pediatric sample. For the adult sample, the proportional odds assumption was violated for the omnibus model (*p* < 0.001), sex (*p* < 0.001), and insurance status (*p* = 0.02). Therefore, given the ordinal structure of the data, a Partial Proportional Odds (PPO) model was conducted for both the pediatric and adult sample. For variables meeting the proportional odds assumption, a single adjusted Odds Ratio (OR) reflecting the likelihood of being in a higher suicide risk category versus a lower suicide risk limitation category was reported. For variables violating the assumption (insurance for the pediatric sample; sex and insurance for the adult sample), ORs were reported for each cumulative threshold (potential or imminent risk versus no risk; imminent risk versus lower risk categories [potential risk or no risk]). Percent increases were calculated so that all values represent higher odds of risk; original ORs are reported in the tables. To identify potential differences in suicide risk across primary diagnosis, a chi-square analysis was run with primary diagnosis and ASQ outcome. Follow up analyses were conducted using Bonferroni-adjusted pairwise comparisons of proportions.

## Results

### Demographic and medical characteristics

Table 1 provides demographic and medical characteristics for both the pediatric and adult samples, as well as ASQ screening outcomes.

**Table 1:** Demographic data and clinical outcomes

	Pediatrics (***N*** = 20,470)	Adults (*N* = 56,629)
	*N* (%)	*N* (%)
Age (Mean, SD)	18.03 (3.64)	49.75 (17.33)
Age groups		
Early adolescence (12–14)	16,200 (79.1)	-
Late adolescence/early adulthood (15–24)	4270 (20.9%)	
Young adulthood (25–34)	-	14,386 (25.4)
Middle adulthood (35–64)		29,423 (52.0)
Older adulthood (65–84)		11,305 (20.0)
Late adulthood (85 +)		1515 (2.7)
Sex		
Male	7341 (35.9)	24,251 (42.8)
Female	13,129 (64.1)	32,378 (57.2)
Race		
Black	14,025 (68.5)	36,783 (65.0)
White	4857 (23.7)	17,313 (30.6)
Hispanic/Latine	61 (0.3)	68 (0.1)
Asian	43 (0.2)	185 (0.3)
Multiracial	253 (1.2)	100 (0.2)
Indian American	55 (0.3)	139 (0.2)
Pacific Islander/Hawaiian	11 (0.1)	28 (0.05)
Other	805 (3.9)	1553 (2.7)
NOT Given	222 (1.1)	453 (0.8)
Insurance		
Government	12,645 (61.8)	26,112 (46.1)
Private	4793 (23.4)	19,678 (34.7)
Uninsured	3032 (14.8)	10,839 (19.1)
Season		
Spring	5181 (25.3)	14,516 (25.6)
Summer	5079 (24.8)	14,024 (24.8)
Fall	5341 (26.1)	14,183 (25.0)
Winter	4869 (23.8)	13,906 (24.6)
Hospital admission unit		
Elective	510 (2.5)	1,243 (2.2)
Emergency	16,107 (78.7)	48,354 (85.4)
Urgent care	3,116 (15.4)	5364 (9.5)
Trauma center	733 (3.6)	1664 (2.9)
Missing	4 (0.02)	4 (0.01)
Primary diagnosis		
Orthopedic	1687 (8.2)	4218 (7.4)
Psychiatric/substance use	1734 (8.5)	5466 (9.7)
Neurological	1013 (4.9)	2806 (5.0)
Women’s health	1041 (5.1)	1725 (3.0)
MVC/GSW/penetrating trauma	1512 (7.4)	2602 (4.6)
Chronic condition	636 (3.1)	2013 (3.6)
General medical	9559 (46.7)	32,463 (57.3)
Missing diagnosis	3288 (16.1)	5336 (9.4)
Discharge location		
Psychiatric facilities	330 (1.6)	285 (0.5)
Non-psychiatric facilities	634 (3.1)	5497 (9.7)
Home	19,506 (95.3)	50,847 (89.8)
ASQ screening outcome		
No risk	19,063 (93.1)	55,126 (97.3)
Potential risk	1089 (5.3)	560 (1.0)
Imminent risk	282 (1.4)	804 (1.4)
Not assessed	36 (0.2)	139 (0.2)

*MVC* motor vehicle collision, *GSW*  gunshot wound, *ASQ*  Ask Suicide-Screening Questions suicide risk screening tool

#### Pediatric sample

In the pediatric sample, mean age was 18.0 years (SD = 3.6; Range = 12–24), with 21% of patients categorized as early adolescence group and 79% as older adolescence/early adulthood. The majority of participants were female (64%) and the sample was racially diverse (69% Black, 24% White, and 7% other races, including Hispanic/Latine, Asian/Pacific Islander, and Indian American). The majority of the patients (62%) used government insurance (e.g., Medicaid/CHIP), 23% used private insurance, and 15% were classified as uninsured.

Regarding presenting primary diagnosis, 56% of pediatric patients were classified as general medical diagnosis, 8% of pediatric patients had a psychiatric or substance use diagnosis, 20% of patients were classified into the other categories (i.e., orthopedic, neurological, motor vehicle collision/gunshot wound, complications related to a chronic condition, or OB/GYN), and 16% of pediatric patients did not have a documented presenting primary diagnosis. The majority (79%) of pediatric patients were admitted to the ED and 21% of patients were admitted to other departments (e.g., urgent care, trauma center). In terms of season, 25% of patients were admitted in the spring, 25% admitted in the summer, 26% admitted in the fall, and 24% admitted in the winter. On the ASQ, 93% of pediatric patients screened as no risk, 5% as potential risk, 1% as imminent risk, and < 1% were not screened.

#### Adult sample

In the adult sample, mean age was 49.8 years (SD = 17.3; Range = 25–113), with 25% of patients classified as young adulthood, 52% as middle adulthood, 20% as older adulthood, and 3% as late adulthood. Regarding sex, 57% of patients were female. Similar to the pediatric sample, racial makeup was diverse with 65% of adult patients being Black, 31% White, and 4% other races. When examining insurance type, 46% of adult patients used government insurance, 35% used private insurance and 19% were uninsured.

Distribution of presenting primary diagnoses was similar to the pediatric sample (see Table 1). The majority (85%) of adult patients were admitted to the ED and 15% were admitted to other departments. With respect to season, 26% of patients were admitted in the spring, 25% admitted in the summer, 25.0% admitted in the fall, and 24% admitted in the winter. On the ASQ, 97% of patients screened as no risk, 1% potential risk, 1% imminent risk, and < 1% were not screened.

### Demographic predictors of ASQ outcome

#### Pediatric sample

Results of the PPO for the pediatric sample are reported in Table 2. Demographic variables (age group, sex, race, insurance type, and season) were entered into the model as predictors. ASQ outcome was treated as an ordinal variable (no risk, potential risk, imminent risk), with higher values indicating greater suicide risk.

**Table 2:** Demographic predictors of suicide risk as measured by the ASQ in the pediatric sample

Factors		*B*	*SE*	*p*-value	aOR	95% confidence interval	
						Lower	Upper
Age groups	Early adolescence (12–14)	0.47	0.064	<0.001	1.60	1.41	1.81
Sex	Male	−0.30	0.062	<0.001	0.74	0.66	0.84
Race	Other/minority	0.16	0.11	0.15	1.17	0.94	1.46
	White	0.66	0.062	< 0.001	1.93	1.71	2.18
Insurance (T1)	Uninsured	−0.50	0.11	< 0.001	0.61	0.49	0.76
	Government	0.099	0.068	0.14	1.10	0.97	1.26
Insurance (T2)	Uninsured	−0.36	0.22	0.094	0.69	0.45	1.06
	Government	−0.12	0.14	0.39	0.89	0.68	1.16
Season	Summer	−0.22	0.078	0.005	0.80	0.69	0.93
	Fall	−0.15	0.076	0.043	0.86	0.74	1.00
	Winter	−0.22	0.080	0.006	0.80	0.69	0.94

Results of partial proportional odds model. As insurance status violated the proportional odds assumption, results are presented for each cumulative threshold (T1 = potential or imminent risk versus no risk; T2 = imminent risk versus no risk or potential risk). The following categories were used as reference categories: age group (late adolescence/early adulthood; 15–24 years), sex (female), race (Black), insurance (private), and season (spring)

*aOR* adjusted odds ratio

**Figure 1:**
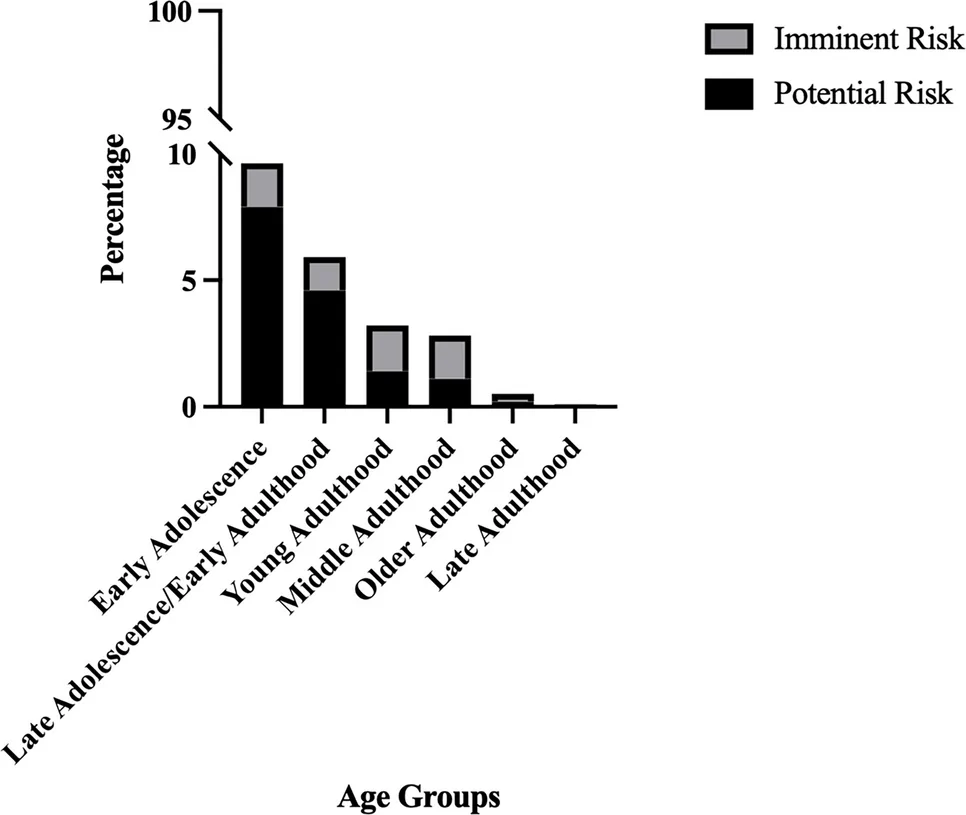
ASQ screening distribution by age group. Stacked bar charts illustrate percentages of potential and imminent risk within each age category

The early adolescence group had 60% higher odds of screening in a higher suicide risk category (*B* = 0.47, *p* < 0.001, OR = 1.60) compared to the late adolescence/early adulthood group. See Fig. 1 for age screening distribution by age group. Compared to males, females had 34% higher odds of screening in a higher suicide risk category (*B* = −0.30, *p* < 0.001, OR = 0.74). White patients had 93% higher odds of screening in a higher suicide risk category (*B* = 0.66, *p* < 0.001, OR = 1.93) compared to Black patients. Threshold-specific effects were examined for insurance. Patients with private insurance had 64% higher odds of screening in a higher risk category (potential or imminent risk versus no risk) compared to youth who were uninsured (*B* = *-*0.50, *p* < 0.001, OR = 0.61). No significant differences were observed between patients with private and government insurance (*p* > 0.05). Regarding season, patients admitted in the spring had significantly higher odds of screening in a higher suicide risk category compared to those admitted in the summer (*B* = *-*0.22, *p* = 0.005, OR = 0.80), fall (*B* = *-*0.15, *p* = 0.04, OR = 0.86), or winter (*B* = *-*0.22, *p* = 0.006, OR = 0.80)*.*

#### Adult sample

Results of the PPO for the adult sample are reported in Table 3. Demographic variables (age group, sex, race, insurance type, and season) were entered into the model as predictors. ASQ outcome was treated as an ordinal variable (no risk, potential risk, imminent risk), with higher values indicating greater suicide risk.

**Table 3:** Demographic predictors of suicide risk as measured by the ASQ in the adult sample

Factors		*B*	*SE*	*p*-value	aOR	95% confidence interval	
						Lower	Upper
Age groups	Middle adulthood (35–64)	−0.29	0.061	< 0.001	0.75	0.67	0.85
	Older adulthood (64–84)	−2.19	0.14	<0.001	0.11	0.084	0.15
	Elderly (85 +)	−3.70	0.71	< 0.001	0.025	0.0061	0.10
Sex (T1)	Male	0.54	0.058	< 0.001	1.71	1.53	1.92
Sex (T2)	Male	0.82	0.076	< 0.001	2.28	1.96	2.64
Race	Other/minority	−0.37	0.17	0.027	0.69	0.50	0.96
	White	0.86	0.057	< 0.001	2.36	2.12	2.64
Insurance (T1)	Uninsured	0.44	0.075	<0.001	1.56	1.35	1.81
	Government	0.45	0.068	< 0.001	1.58	1.38	1.80
Insurance (T2)	Uninsured	0.54	0.10	< 0.001	1.71	1.42	2.07
	Government	0.58	0.089	< 0.001	1.78	1.50	2.12
Season	Summer	0.22	0.079	0.005	1.25	1.07	1.46
	Fall	0.15	0.080	0.056	1.16	1.00	1.36
	Winter	0.15	0.081	0.071	1.16	0.99	1.35

Results of partial proportional odds model. As sex and insurance status violated the proportional odds assumption, results are presented for each cumulative threshold (T1 = potential or imminent risk versus no risk; T2 = imminent risk versus no risk or potential risk). The following categories were used as reference categories: age group (young adulthood; 24–34 years), sex (female), race (Black), insurance (private), and season (spring)

*aOR* adjusted odds ratio

The young adulthood group had significantly higher odds of screening in a higher suicide risk category (all *p* values < 0.05) compared to the middle adulthood, older adulthood, and late adulthood group. Threshold-specific effects were examined for sex. Compared to females, males had 71% higher odds of screening as potential or imminent risk versus no risk (*B* = 0.53, *p* < 0.001, OR = 1.71) and 128% higher odds of screening as imminent risk versus lower risk categories (*B* = 0.82, *p* < 0.001, OR = 2.28). White patients had 137% higher odds of screening in a higher suicide risk category compared to Black patients (*B* = 0.86, *p* < 0.001, OR = 2.36). Black patients had 45% higher odds of suicide risk compared to patients in the Other racial category (*B* = −0.37, *p* = 0.028, OR = 0.69). Threshold-specific effects were also examined for insurance, and results indicated that compared to patients with private insurance, those with government insurance had 58% higher odds of screening as potential or imminent risk versus no risk (*B* = 0.45, *p* < 0.001, OR = 1.58) and 78% higher odds of imminent risk versus lower risk categories (*B* = 0.58, *p* < 0.001, OR = 1.78). Additionally, patients who were uninsured had 56% higher odds of screening as potential or imminent risk (*B* = 0.44, *p* < 0.001, OR = 1.56) and 71% higher odds of screening as imminent risk (*B* = 0.53, *p* < 0.001, OR = 1.71) compared to those with private insurance. Regarding season, adult patients admitted in the summer had 25% higher odds of a higher suicide risk category compared to those admitted in the spring (*B* = 0.22, *p* = 0.005, OR = 1.25).

### Difference in ASQ outcomes by primary diagnosis

#### Pediatric sample

See Table 4 for ASQ screening rates for primary diagnosis categories in the pediatric and adult sample. Chi-square analyses indicated proportional differences in screening risk outcomes across primary diagnosis categories, χ2 (12) = 4520.30, *p* < 0.001. Post-hoc Bonferroni-adjusted comparisons revealed that the psychiatric/substance use category had a significantly higher proportion of potential (32%) and imminent risk (15%) screenings compared to the other primary diagnosis categories (*p* < 0.05).

**Table 4:** ASQ screening outcomes by primary diagnosis

	Pediatric			Adult		
	Percentage (%)			Percentage (%)		
	No risk	Potential risk	Imminent risk	No risk	Potential risk	Imminent risk
*Diagnosis*						
Orthopedic	97.6	2.3	0.06	99.6	0.2	0.2
Psychiatric/substance use	53.5	31.7	14.8	80.7	6.1	13.2
Neurological	95.4	4.5	0.10	99.4	0.4	0.2
Women’s health	97.8	2.1	0.10	99.4	0.6	0.0
MVC/GSW/penetrating trauma	98.1	1.5	0.3	99.1	0.6	0.3
Chronic condition	95.8	4.2	0.0	99.5	0.4	0.2
General medical	96.5	3.3	0.2	97.5	1.0	1.6

*MVC* motor vehicle collision, *GSW*  gunshot wound, *ASQ*  Ask Suicide-Screening Questions suicide risk screening tool

In order to determine differences in ASQ outcome across diagnoses when psychiatric illnesses were not considered, an additional chi-square analysis was run without participants in the psychiatric/substance use category. The overall model remained significant, χ2 (10) = 35.81, *p* < 0.001, indicating that suicide risk significantly differed between medical primary diagnoses in the pediatric sample. Post-hoc Bonferroni-adjusted comparisons indicated that the neurological (5%), chronic condition (4%), and general medical condition (3%) had a significantly higher proportion of potential risk screenings compared to the women’s health (2%), MVC/GSW/Trauma (2%), and orthopedic (2%) categories. There were no significant proportional differences between primary diagnosis categories for imminent risk.

#### Adult sample

In the adult sample, chi-square analyses demonstrated proportional differences in screening risk outcomes across primary diagnosis categories, χ2 (12) = 7097.70, *p* < 0.001. Post-hoc Bonferroni-adjusted pairwise comparisons revealed that the psychiatric/substance use category had a significantly higher proportion of potential (6%) and imminent risk (13%) screenings compared to the other primary diagnosis categories (*p* < 0.05). When analyses were run excluding patients in the psychiatric/substance use category, the model remained significant, χ2 (10) = 18.51, *p* = 0.047. However, post-hoc Bonferroni-adjusted comparisons indicated that there were no proportional differences in screening as potential or imminent risk across primary diagnosis categories.

## Discussion

This study sought to build upon prior research by examining suicide risk rates as assessed through universal hospital screening with the ASQ.^13,16–20^ The authors aimed to identify demographic predictors of suicide risk outcomes and conducted a novel analysis exploring how risk may differ across medical diagnoses. The pediatric sample (6%) exhibited higher rates of risk compared to the adult sample (2%). Current results highlight the importance of universal suicide risk screening in hospital settings, particularly in pediatric populations. Within the pediatric sample, early adolescents (12–14 years old) exhibited significantly higher likelihood of greater suicide risk compared to late adolescents/early adulthood (15–24 years old). These results align with prior longitudinal research illustrating an increase in psychopathology (e.g., depression and anxiety) in early-adolescent females, and notably, that depression and anxiety symptoms in early adolescence predict suicide attempts into adulthood.^23^ Given the increase in younger adolescents on social media in the last decade and the associated psychopathology with social media and youth, this study’s findings further bolster the need for screening in pediatric settings.^24^ Additionally, in the adult sample, young adults- and to a lesser extent, middle-aged adults, also demonstrated an elevated likelihood of suicide risk. These trends are consistent with recent data showing that suicidal ideation in young adults has significantly increased over the past decade.^25^

In terms of sex differences, consistent with prior research, females in the pediatric sample had a higher likelihood of suicide risk, while in the adult sample, males had a higher likelihood of suicide risk. ^17^ Also consistent with prior studies, Black and other minority youth and adults had a lower likelihood of reporting suicide risk compared to White patients.^26,27^ Liu and colleagues hypothesized that these differences in reported suicide risk may be because minority patients (e.g., Black, Latine) are less likely to disclose mental health symptoms (e.g., suicidal ideation) due to stigma or language. Further noting discrepancies in mental health care, a study utilizing data from the National Survey on Drug Use and Health found that racial/ethnic minority adolescents were less likely to receive inpatient treatment for suicidal ideation and behavior compared to White adolescents.^28^ Thus, continued research on culturally sensitive measures for racial/ethnic minority patients remains critical to ensure equitable detection and intervention.

Youth who were uninsured had significantly lower odds (39%) of being screening in a higher risk category compared to patients with private insurance. Based on 2023 data specific to the region this study was conducted in, 6% of children were uninsured, with rates of being uninsured particularly high in minority youth (e.g., 15% of Hispanic/Latine children; 10% of multiracial children).^29^ In the current sample, 82% of children who were uninsured were of minority racial status. Thus, these results are consistent with aforementioned research suggesting that minority populations may report or disclose suicide risk less often than their White counterparts. In the current adult sample, adults with government insurance or no insurance had significantly higher odds (56% to 78%) of reporting both potential and imminent suicide risk compared to private insurance. These findings are consistent with prior research in adults demonstrating increased rates of suicidal ideation and suicide attempts in patients with Medicaid or no insurance.^30,31^

As anticipated, patients with psychiatric or substance use diagnoses had the highest rates of suicide risk (pediatric sample: 47%; adult sample: 19%). In the adult sample, there were no significant differences in the rates of potential and imminent risk among the medical (non-psychiatric/substance use disorder) diagnosis categories. In the pediatric sample, the neurological (6%), chronic condition (4%), and general medical condition (3%) categories had significantly higher reported rates of risk compared to the other primary diagnosis categories. These results support findings from a 2024 cohort study reporting that out of nine medical categories, individuals with gastrointestinal conditions, cancer, and hematological conditions had the highest risk of suicide.^32^ Prior research suggests, that while there is likely etiological heterogeneity for suicide risk in pediatric patients with chronic conditions, early identification of suicidal behavior may help patients receive appropriate interventions.^33^ Additionally, hospital suicide screening followed by targeted intervention has been shown to be effective in significantly reducing suicide attempts compared to treatment as usual.^34,35^

### Limitations

This study has several limitations. Foremost, given that this study was conducted at an academic medical center in the Southeast, these results may not be generalizable to other regions with differing demographic characteristics (e.g., racial demographics). Additionally, the patient population in this study may differ from those in community hospitals or rural healthcare systems, which may limit the external validity of our findings. Another limitation is that this study describes results of the first year of an institution-wide quality improvement projects, and as such, data are subject to clinical constraints. Specifically, 16% of pediatric patients and 9% of adult patients were not provided a primary diagnosis at the time of ASQ screening, introducing non-random missing data biases into these statistical analyses. Additionally, due to this study being a quality improvement project, a limited number of demographic and medical variables were captured and examined as predictors of ASQ screening outcomes. For instance, individuals of sexual and gender minority status are known to have higher rates of suicide risk, and these characteristics were not captured within this study.^36^ Despite these limitations, this study has several notable strengths. In particular, missing ASQ screening data were minimal (< 1% in both the pediatric and adult samples), indicating excellent implementation of ASQ screening at a large scale. In addition, this present sample was racially (65–69% Black, 24–31% White) and economically (46–62% government insurance, 23–35% private insurance) diverse and is one of the first to examine implementation of universal ASQ screening across both pediatric and adult hospital settings.

## Implications for Behavioral Health

This study highlights the success and importance of universal ASQ suicide screening across both pediatric and adult settings at a Southeastern academic medical center. Results revealed a consistent pattern of elevated suicide risk among early-adolescent patients (12–14 years old). Young females, younger and middle-aged adults, male adults, and adults with government insurance or no insurance also exhibited increased suicide risk. Youth with chronic conditions, neurological conditions, and general medical conditions exhibited higher rates of suicide risk but otherwise risk was relatively consistent across medical diagnosis categories. These findings provide evidence that standardized universal suicide screening in both pediatric and adult hospital settings can be effective in identifying patients at elevated suicide risk to initiate appropriate interventions for suicide prevention. Additionally, results from this study demonstrate the utility of the ASQ in mixed settings, including both across pediatric and adult populations and in multiple settings across a large hospital. Expanding and standardizing suicide risk screening across health care systems is an important step to provide early identification of suicide risk and to ensure subsequent intervention.

Findings from this present study also highlight the importance of considering contextual factors when conducting suicide risk assessment and intervention. Providers should consider access to care, socioeconomic status, and cultural factors that may influence how patients disclose mental health symptoms. Thus, culturally informed approaches for screening suicide risk and subsequent intervention are important to assess for suicide risk across diverse populations. Future studies are needed to better characterize suicide risk in youth from minoritized groups (e.g., race, sexuality, gender), to better understand predictive factors at a population level, and to improve preventative intervention efforts to reduce suicide risk.

## Data Availability

The data for this project are available from the corresponding author upon reasonable request.
